# Prevascularization of dermal substitutes with adipose tissue-derived microvascular fragments enhances early skin grafting

**DOI:** 10.1038/s41598-018-29252-6

**Published:** 2018-07-20

**Authors:** Florian S. Frueh, Thomas Später, Christina Körbel, Claudia Scheuer, Anna C. Simson, Nicole Lindenblatt, Pietro Giovanoli, Michael D. Menger, Matthias W. Laschke

**Affiliations:** 1 Institute for Clinical and Experimental Surgery, Saarland University, 66421 Homburg/Saar, Germany; 2 Division of Plastic Surgery and Hand Surgery, University Hospital Zürich, University of Zürich, 8091 Zürich, Switzerland

## Abstract

Split-thickness skin grafts (STSG) are still the gold standard for the treatment of most skin defects. Hence, there is an ongoing need to improve this procedure. For this purpose, we herein analyzed dermal matrices seeded with adipose tissue-derived microvascular fragments (ad-MVF) in a bradythrophic wound model. In additional experiments, the matrices were covered with autologous STSG 10 days after implantation. Green fluorescence protein (GFP)^+^ ad-MVF were isolated from C57BL/6-Tg(CAG-EGFP)1Osb/J mice and seeded onto collagen-glycosaminoglycan matrices. Non-seeded and prevascularized matrices were implanted into full-thickness skin defects on the skull of CD1 nu/nu mice for 21 days. Vascularization, lymphangiogenesis and incorporation of the matrices were analyzed using photo-acoustic imaging, trans-illumination stereomicroscopy, histology, and immunohistochemistry. The survival rate of STSG was assessed by planimetry. After 21 days, the density of microvascular and lymphatic networks was significantly higher in prevascularized matrices when compared to controls. This was associated with an improved implant integration. Moreover, prevascularization with ad-MVF allowed successful autologous skin grafting already at day 10, while coverage of non-seeded controls at day 10 resulted in STSG necrosis. In conclusion, ad-MVF represent powerful vascularization units. Seeded on dermal substitutes, they accelerate and enhance the healing of full-thickness skin defects and allow early coverage with STSG.

## Introduction

Full-thickness skin defects with impaired vascularization are a reconstructive challenge. These wounds are typically encountered when treating diabetic ulcers, burn injuries, or after tumor resection^1^. In the United States, around 6.5 million patients are affected by chronic wounds and the burden is growing due to an aging population and rising incidence of diabetes and obesity^2^. Thus, there is an on-going need to develop innovative wound-healing strategies.

The treatment of bradytrophic wounds with split-thickness skin grafts (STSG) alone is rarely successful, because the underlying tissues do not exhibit a sufficient vascularization capacity. Moreover, STSG without dermal support are prone to extensive scarring and contraction^3^. To overcome these problems, bioengineered dermal substitutes have been introduced. The FDA-approved Integra (Integra Life Sciences, Plainsboro, NJ, USA) consists of a collagen-glycosaminoglycan matrix with a silicone pseudo-epidermis and is frequently used in clinical practice^4^. However, microvascular network formation within the matrix requires up to 3 weeks before STSG coverage can be performed^5^. This vascularization kinetics crucially determines the risk of wound infection, which is elevated as long as the physiological barrier of the skin is not re-established^6^.

The vascularization of dermal substitutes can be improved by means of angiogenic or prevascularization approaches^7^. Prevascularization based on the seeding of adipose tissue-derived microvascular fragments (ad-MVF) is particularly suitable for intraoperative one-step procedures and, therefore, a promising strategy for future clinical application^8,9^. Ad-MVF are functional vessel segments that rapidly reassemble into microvascular networks after transplantation^10^. Recently, we demonstrated that the prevascularization of Integra with ad-MVF enhances incorporation and epithelialization as well as the development of microvascular and lymphatic networks within the matrix^11^. These results led to the hypothesis that ad-MVF seeding may also accelerate the integration and vascularization of Integra in bradytrophic wounds, which may then allow early skin grafting.

To test this hypothesis, we used a full-thickness skin defect model on the skull of mice, mimicking bradytrophic wounds after tumor resection on the human scalp. Implanted non-seeded and prevascularized Integra matrices were assessed for 21 days using stereomicroscopy, photo-acoustic imaging, histology and immunohistochemistry. In a subsequent proof-of-principle experiment, coverage with autologous STSG was performed 10 days after Integra implantation and the grafts’ survival was analyzed.

## Results

### Full-thickness skin defect model

**Figure 1:**
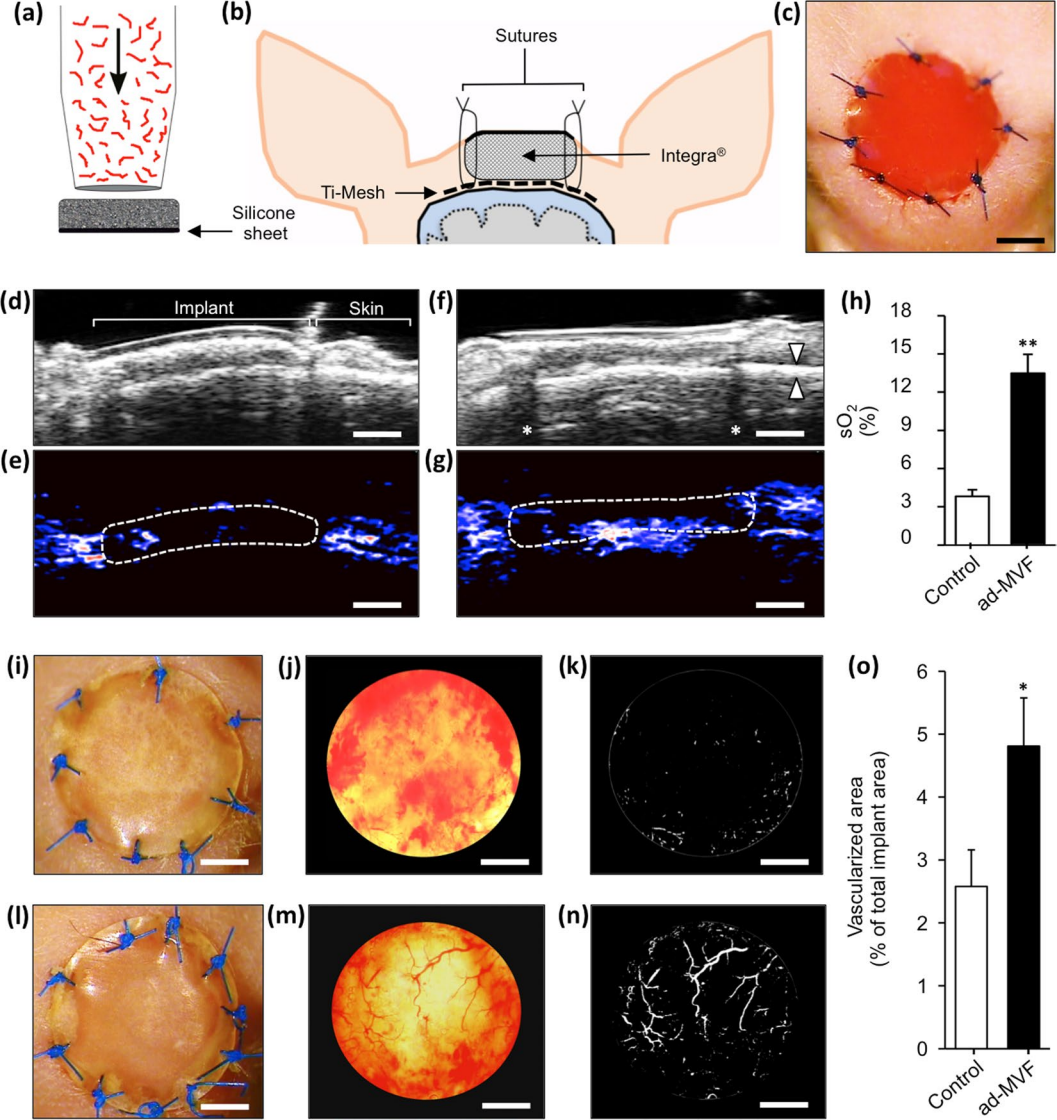
Animal model, photo-acoustic imaging and trans-illumination stereomicroscopy. (**a**–**c**) After the seeding process (**a**), the matrices were implanted into full-thickness skin defects on the skull of CD1 nu/nu mice (**b** and **c**). (**d**–**g**) B-mode ultrasound (**d** and **f**) and OxyHemo-mode photo-acoustic imaging (**e** and **g**) of non-seeded (**d** and **e**) and prevascularized (**f** and **g**) Integra 21 days after implantation. Red areas = high oxygenation, blue areas = low oxygenation, arrowheads in **f** = frontal calvaria, asterisks in **f** = dorsal acoustic attenuation (sutures), broken line in **e** and **g** = implants. (**h**) Quantification of sO_2_ (%). Mean ± SEM, n = 8, **P < 0.001 vs. non-seeded control. (**i**–**n**) Epi-illumination (**i** and **l**) and trans-illumination (**j** and **m**) microscopy with digital segmentation images (**k** and **n**) of non-seeded (**i**–**k**) and prevascularized (**l**–**n**) Integra. (**o**) Quantification of vascularized area (% of total implant area). Mean ± SEM, n = 8, *P < 0.05 vs. non-seeded control. Scale bars: **c** = 2.5 mm, **d**–**g** = 1.8 mm, **i**–**n** = 2 mm.

Ad-MVF were isolated from green fluorescent protein (GFP)^+^ donor mice and seeded onto Integra as previously described^11,12^ (Fig. 1a). Non-seeded Integra served as control. To analyze the vascularization and incorporation of the matrices, full-thickness skin defects were prepared on the skull of CD1 nu/nu mice. For this purpose, we modified the head punch model^13,14^ and prepared an 8 mm skin defect on the crown of the skull. The periosteum was resected to create a poorly vascularized, bradytrophic wound bed. To ensure implant immobilization, a titanized mesh was placed on the bone overlapping the wound edges according to a published model^15^. Subsequently, non-seeded and ad-MVF-seeded (prevascularized) Integra matrices were implanted and secured with sutures (Fig. 1b and c).

### Photo-acoustic imaging and trans-illumination stereomicroscopy

Photo-acoustic imaging was performed to quantify *in situ* the oxygen saturation within the neo-dermis of non-seeded and prevascularized Integra 21 days after implantation. This approach revealed that prevascularized matrices were characterized by significantly higher oxygenation levels than non-seeded controls (Fig. 1d–h).

In a next step, the implants were excised and assessed using trans-illumination stereomicroscopy to objectify microvascular network formation. In line with the photo-acoustic results, this analysis revealed a significantly reduced vascularization in non-seeded controls when compared to matrices seeded with ad-MVF (Fig. 1i–o).

### Histology and immunohistochemistry

**Figure 2:**
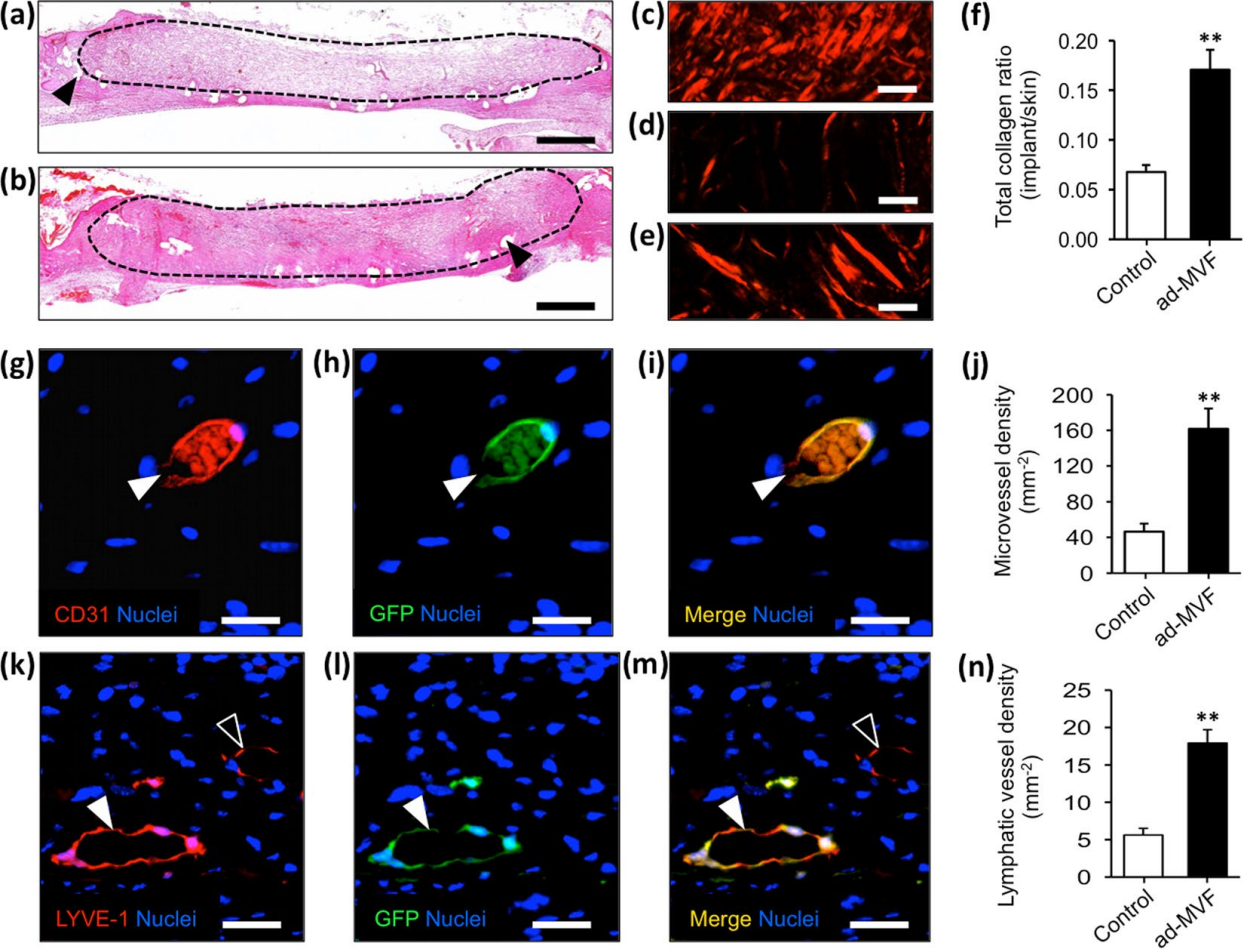
Histology and immunohistochemistry. (**a** and **b**) HE-stained sections of non-seeded (**a**) and prevascularized (**b**) implants. Broken line = implant border, arrowheads = mesh fibers. (**c**–**e**) Polarized light microscopy of Sirius red-stained sections of normal skin (**c**), non-seeded (**d**) and prevascularized (**e**) Integra. (**f**) Quantification of total collagen ratio (implant/skin). Mean ± SEM, n = 8, **P < 0.001 vs. non-seeded control. (**g**–**i** and **k**–**m**) Immunohistochemical staining of microvessels (**g**–**i**, white arrowhead = CD31^+^/GFP^+^ microvessel) and lymphatic vessels (**k**–**m**, white arrowhead = LYVE-1^+^/GFP^+^ lymphatic vessel, empty arrowhead = LYVE-1^+^/GFP^−^ lymphatic vessel) within prevascularized Integra 21 days after implantation. (**j** and **n**) Quantification of microvessel density (mm^−2^) and lymphatic vessel density (mm^−2^). Mean ± SEM, n = 8, **P < 0.001 vs. non-seeded control. Scale bars: **a** and **b** = 800 µm, **c**–**e** = 20 µm, **g**–**i** = 20 µm, **k**–**m** = 30 µm.

Histological analyses of the implants showed that non-seeded Integra was characterized by a low cellular infiltration (Fig. 2a). In contrast, the neo-dermis of prevascularized Integra exhibited a dense granulation tissue (Fig. 2b). Accordingly, the integration of the prevascularized matrices was markedly enhanced, as indicated by significantly more mature Sirius red stained collagen fibers when compared to non-seeded controls (Fig. 2c–f).

Immunohistochemical detection of the endothelial cell marker CD31 demonstrated that the prevascularized matrices exhibited a 3.5-fold higher microvessel density 21 days after implantation when compared to non-seeded controls (Fig. 2g–j). GFP/CD31 co-staining further revealed that > 95% of the microvessels within the prevascularized implants were GFP^+^. Moreover, the lymphatic vessel density of prevascularized implants was markedly increased (Fig. 2k–n). However, compared to the microvasculature, a lower fraction of lymphatic vessels exhibited a GFP^+^ signal (88 ± 1%).

### Autologous skin grafting

**Figure 3:**
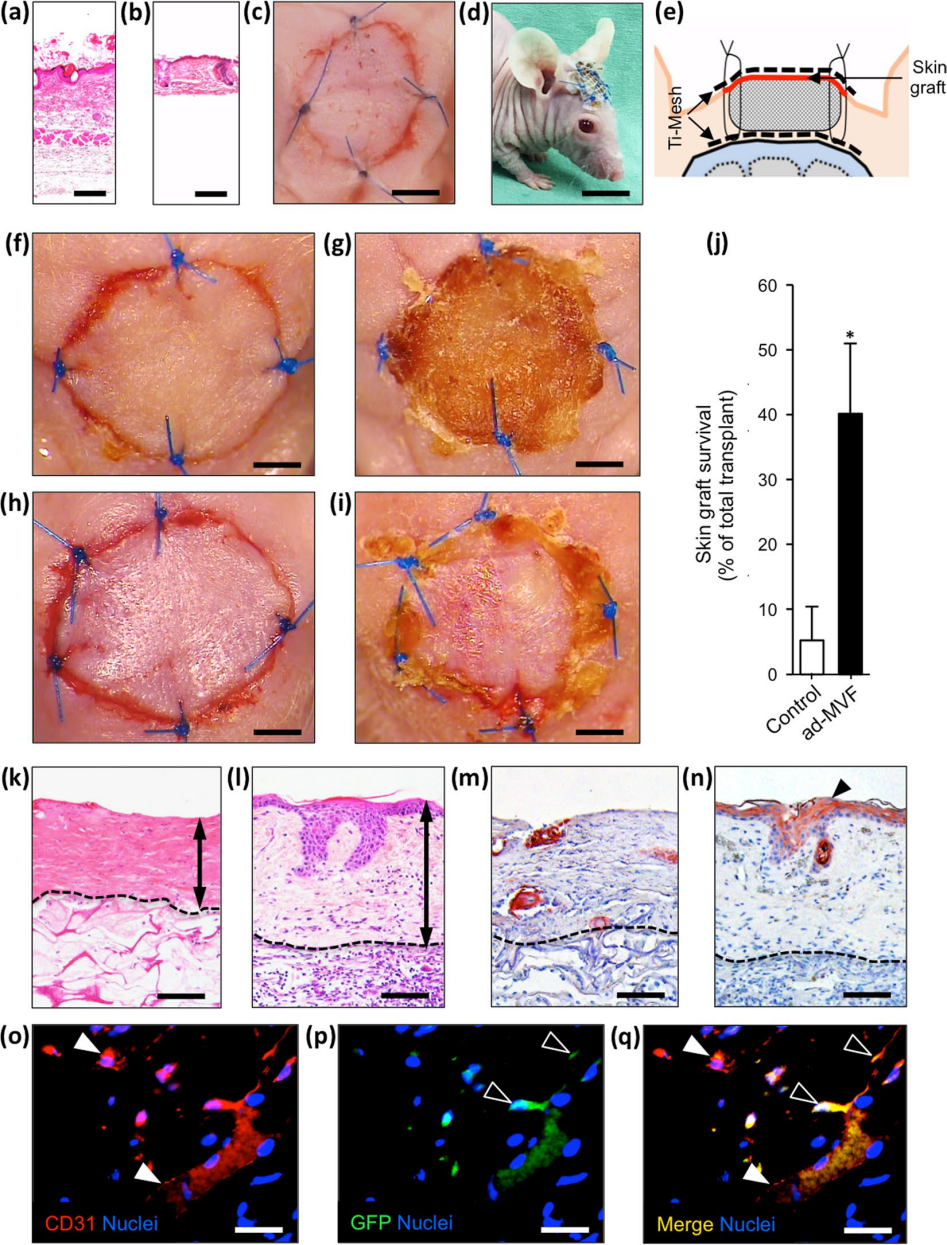
Autologous skin grafting. (**a**–**e**) HE-stained sections of a skin graft before (**a**) and after (**b**) defatting. The autologous grafts are transferred onto implanted Integra and are secured with sutures, a sterile plastic dressing (**c**) and a titanized mesh (**d** and **e**). (**f**–**i**) Stereomicroscopy of non-seeded (**f** and **g**) and prevascularized (**h** and **i**) implants immediately (**f** and **h**) and 5 days (**g** and **i**) after skin grafting. (**j**) Quantification of skin graft survival (% of total transplant) 5 days after transplantation. Mean ± SEM, n = 6, *P < 0.05 vs. non-seeded control. (**k** and **l**) HE-stained sections after skin grafting of non-seeded (**k**) and prevascularized (**l**) Integra. Double arrows = graft thickness. (**m** and **n**) Immunohistochemical detection of cytokeratin^+^ epithelium (**n**, arrowhead) after grafting of a non-seeded (**m**) and prevascularized (**n**) matrix. (**o**–**q**) Immunohistochemical detection of CD31^+^ microvessels (**o** and **q**, white arrowheads) within a skin graft 5 days after transplantation on prevascularized Integra. CD31/GFP co-staining reveals CD31^+^/GFP^+^ cells (**p** and **q**, empty arrowheads) involved in microvessel formation. Scale bars: **a** and **b = **120 µm, **c** = 3 mm, **d** = 9 mm, **f**–**i** = 1.8 mm, **k**–**n** = 80 µm, **o**–**q** = 35 µm.

In additional experiments, we analyzed the survival of autologous STSG, which were transplanted onto non-seeded and prevascularized Integra 10 days after implantation. Full-thickness skin grafts (Fig. 3a) were harvested from the groin of CD1 nu/nu mice and defatted to imitate STSG (Fig. 3b). The STSG were then transplanted onto the matrices and secured with sutures. To protect the STSG from exsiccation, a sterile plastic dressing (Fig. 3c) and a second titanized mesh were fixed to the previously implanted mesh (Fig. 3d and e).

### Planimetric and histological analysis of STSG

The survival of the STSG was assessed by planimetric analyses 5 days after transplantation. STSG on prevascularized Integra exhibited a survival rate of 40 ± 11%. In contrast, skin grafting on non-seeded controls resulted in a significantly lower transplant survival of 5 ± 5% (Fig. 3f–j).

Histological analyses revealed that dermal thickness and hierarchical integrity of the STSG were more preserved in the prevascularized group when compared to controls (Fig. 3k and l). Moreover, the transplantation of STSG on prevascularized implants also resulted in a thicker cytokeratin^+^ epidermis (Fig. 3m and n). Finally, immunohistochemical GFP/CD31 co-staining was performed for a qualitative assessment of cell migration into the STSG. Of interest, we detected a few GFP^+^ cells, which were incorporated into the CD31^+^ endothelium of individual microvessels (Fig. 3o–q).

## Discussion

The treatment of bradytrophic skin defects is a major challenge. In the last decade, skin tissue engineering has evolved dramatically with promising approaches to manage difficult wounds^16^. In fact, preconditioned hydrogels^17–19^, microporous gels^20^ or engineered cell sheets^21,22^ exhibit a tremendous wound healing potential in the preclinical setting. However, the majority of these techniques involves complex *in vitro* steps and, thus, is not suitable for intraoperative one-step procedures. Hence, straightforward strategies need to be developed.

We have recently introduced ad-MVF-seeded Integra as a powerful strategy to treat full-thickness skin defects^11^. In the present study, we further evaluated this approach in a murine wound model with early STSG coverage. For this purpose, we used hairless CD1 nu/nu mice, because they enabled the application of compressive wound dressings and photo-acoustic imaging without the need for repetitive mechanical and chemical skin depilation, which may have markedly affected the incorporation of the implanted matrices and the engraftment of STSG. In addition, these immunoincompetent animals allowed the transplantation of ad-MVF from GFP^+^ donor mice without inducing a rejection reaction. Hence, it was possible to identify GFP^+^ ad-MVF-derived and GFP^-^ host-derived blood and lymphatic vessels within the implanted matrices. On the other hand, it should be considered that CD1 nu/nu mice lack a thymus and, thus, are unable to produce T-cells. Importantly, epidermal and dermal T-cells are crucially involved in the regulation of murine wound healing by local production of epithelial growth factors and inflammatory cytokines^23–26^. Moreover, human epidermal T-cells have been shown to contribute to the effective healing of acute wounds and are functionally defective in patients with chronic wounds^27,28^. Accordingly, it can be assumed that the implantation of ad-MVF-seeded matrices into immunocompetent animals might have even resulted in an improved vascularization and incorporation of the implants when compared to the herein reported results. This further underlines the high potential of ad-MVF for applications in regenerative medicine.

To create full-thickness skin defects with a poorly vascularized wound bed, we combined two mouse models^13,15^. Even though the literature negates wound contraction in the head punch model^29^ we observed implant loss without additional stabilization. Hence, we used a titanized mesh to secure the dermal substitute. Importantly, we did not observe significant foreign body reactions around the mesh. In our model, the surrounding skin margin represented the only site for ad-MVF inosculation or angiogenic ingrowth.

Photo-acoustic imaging and trans-illumination stereomicroscopy showed a significantly higher oxygenation and vascularization in the prevascularized matrices when compared to non-seeded controls. Accordingly, we also measured an increased microvessel density. Moreover, >95% of the microvessels within the prevascularized implants were GFP^+^, indicating that they originated from the seeded ad-MVF. This finding is important, because in contrast to previous studies^11,30,31^ we herein used the double-layer Integra, which is covered with a silicone sheet. Hence, ad-MVF could only be seeded on the bottom side of the matrices and were implanted in direct contact to the bone of the mouse skull. Nonetheless, GFP^+^ microvessels were homogeneously distributed in deep and superficial layers of the matrices at day 21 after implantation. This suggests that the ad-MVF even survive in an initially poorly oxygenated environment and rapidly reassemble into new microvascular networks, most probably driven by hypoxia-induced secretion of angiogenic growth factors.

The two-staged treatment of bradytrophic skin defects with dermal substitutes and STSG is a common procedure in clinical practice. To further unravel the potential of prevascularization with ad-MVF, we assessed the fate of autologous STSG in a proof-of-principle experiment. The application of STSG in mice is technically demanding due to the small animal size and thin skin. Because conventional STSG harvesting is not feasible, we microsurgically removed the panniculus carnosus muscle and the deep dermal layer of excised skin samples. This resulted in skin grafts similar to STSG. The compression of these grafts was the major prerequisite for successful engraftment. For this purpose, we fixed them with a titanized mesh, which was well tolerated by the animals. No graft was lost due to manipulation. In pilot experiments, we performed STSG coverage already at day 0, i.e. immediately after the implantation of Integra. However, this one-step procedure was not effective with complete graft necrosis. This may be explained by the fact that the onset of blood perfusion in ad-MVF-derived microvascular networks requires 3–6 days^11^. Consequently, we decided to cover the implants at day 10. At this time point, the neo-dermis of the prevascularized matrices was stable and allowed STSG coverage. Importantly, graft survival in the prevascularized group was significantly improved when compared to non-seeded controls.

Previous experiments with full-thickness skin grafts in the dorsal skinfold chamber model revealed that blood vessel ingrowth from the wound bed crucially contributes to graft revascularization^32,33^. In these studies, reperfusion was established ~3 days after transplantation and subsequently, an angiogenic response was detected within the skin grafts’ capillaries. Of interest, angiogenesis started in the center of the grafts, indicating that the hypoxic stimulus may be most prominent in this area^33^. Accordingly, our study also revealed graft survival mainly in the center of the transplants. This finding was consistent in all vital grafts and supports the theory of hypoxia-induced angiogenesis in central parts of STSG.

Ad-MVF are a rich source of regenerative cells. In fact, they contain a relevant fraction of Sca-1^+^/VEGFR-2^+^ endothelial progenitor cells^10,34^. These cells boost wound revascularization and reoxygenation in mice^35^. To analyze cellular interactions between the wound bed and the skin grafts, co-staining with CD31/GFP was performed. Indeed, we could show that GFP^+^ cells incorporated into the CD31^+^ endothelium of individual microvessels within the STSG. Hence, it can be assumed that the revascularization of the grafts was achieved by external inosculation, i.e. the outgrowth of the grafts’ microvessels into the surrounding skin^36^, and by the ingrowth of GFP^+^ ad-MVF-derived vascular sprouts from the prevascularized matrices. However, this conclusion may not completely explain the advantageous effect of prevascularized Integra on the survival rate of STSG. As indicated by photo-acoustic imaging, the prevascularized matrices exhibited a significantly higher oxygen level. Consequently, we speculate that the ~200 µm thick skin grafts on ad-MVF-enhanced neo-dermis were supplied by diffusion with life-sustaining oxygen and growth factors to bridge the critical 48–72 h after implantation.

Beside a sufficient vascularization, lymphatic vessel formation is essential to re-establish skin function. Dermal lymphatic vessels are crucially involved in the regulation of tissue fluid homeostasis and immune cell trafficking^37^. Adipose tissue-derived stem cells have shown potential to support lymphangiogenesis *in vitro*^38^ and *in vivo*^39^. To assess lymphangiogenic effects within non-seeded and prevascularized Integra, we quantified the lymphatic vessel density 21 days after implantation. We found that prevascularized Integra contained >3-fold more lymphatic vessels than non-seeded controls. We hereby confirm the recently observed lymphangiogenic effect of ad-MVF^11^. Finally, lymphatic vessels play an important role in the engraftment of STSG. However, to observe lymphatic anastomoses between STSG and the wound bed, up to 14 days observation after grafting are required^18^. Accordingly, within the short observation period of 5 days, we were not able to detect lymphatic interconnections in the present study.

In conclusion, this study indicates that ad-MVF represent powerful vascularization units. Seeded on dermal substitutes, they enhance the vascularization and incorporation of dermal substitutes in skin defects exhibiting a bradytrophic wound bed. In addition, they allow the early coverage of Integra with STSG. Hence, they may markedly contribute to shorten the time frame needed for future skin reconstruction and, thus, to reduce the infection risk and hospitalization times for patients.

## Methods

### Animals

Full-thickness skin defects were prepared in CD1 nu/nu mice (age: ~3 months, body weight: 30–32 g). Ad-MVF were isolated from C57BL/6-Tg(CAG-EGFP)1Osb/J mice (age: 7–12 months, body weight: >30 g; The Jackson Laboratory, Bar Harbor, ME, USA)^40^. The animals were housed under a 12 h light/dark cycle and received water and standard food pellets (Altromin, Lage, Germany) *ad libitum*.

All experiments were approved by the local governmental animal care committee (Landesamt für Verbraucherschutz, Saarbrücken, Germany; permit number: 25/2016) and conducted in accordance with the European legislation on the protection of animals (Directive 2010/63/EU) and the National Institutes of Health (NIH) guidelines on the care and use of laboratory animals (NIH publication #85–23 Rev. 1985).

### Isolation of ad-MVF

Epididymal fat pads were harvested from GFP^+^ donor mice, transferred into 10% Dulbecco’s modified eagle medium (DMEM; 100 U/mL penicillin, 0.1 mg/mL streptomycin; Biochrom GmbH, Berlin, Germany), and washed with phosphate-buffered saline (PBS; Biochrom GmbH). The fat was mechanically minced and digested for 10 min with collagenase NB4G (0.5 U/mL; Serva Electrophoresis GmbH, Heidelberg, Germany) while stirring under humidified atmospheric conditions (37 °C, 5% CO_2_). The digestion was neutralized with PBS supplemented with 20% fetal calf serum (FCS; Biochrom GmbH) and the cell-vessel suspension was incubated for 5 min at 37 °C. After removal of fat supernatant, the remaining cell-vessel suspension was filtered through a 500 μm mesh (pluriStrainer; pluriSelect Life Science, Leipzig, Germany) and centrifuged for 5 min at 600 × g to obtain a pellet.

### Seeding of Integra

Dermal substitutes (diameter: 8 mm) were cut out of Integra Dermal Regeneration Template (Integra Life Sciences, Ratingen, Germany) with a biopsy punch. For each recipient mouse, a pellet containing ~ 40,000 GFP^+^ ad-MVF isolated from 1 mL fat tissue was mixed with 20 µL 0.9% NaCl and was seeded on the collagen-glycosaminoglycan surface of Integra with a 20 µL precision pipette (Eppendorf, Wesseling-Berzdorf, Germany). The same procedure without ad-MVF was performed for non-seeded implants of the control group.

### Full-thickness skin defect model

CD1 nu/nu mice were anesthetized by intraperitoneal injection of ketamine (75 mg/kg; Ursotamin, Serumwerk Bernburg AG, Bernburg, Germany) and xylazine (15 mg/kg; Rompun, Bayer, Leverkusen, Germany) and were placed under a stereomicroscope (Leica M651, Wetzlar, Germany). Subsequently, a skin defect on the crown of the skull was prepared using an 8 mm biopsy punch and the periosteum was resected with microsurgical instruments. A titanized mesh (TiMesh; pfm medical AG, Köln, Germany) was placed on the bone overlapping the wound edges. Next, non-seeded and prevascularized Integra matrices were implanted and secured with interrupted 6/0 monofilament. Postoperative analgesia was provided for 3 days with tramalhydrochloride (40 mg/100 mL drinking water; Grünenthal GmbH, Aachen, Germany).

For the transplantation experiments, full-thickness skin grafts were excised from the right groin of anesthetized CD1 nu/nu mice and defatted under a stereomicroscope. The STSG were transplanted onto the Integra matrices and secured with interrupted 6/0 monofilament. Finally, a sterile plastic dressing and a second titanized mesh were fixed to the previously implanted mesh with interrupted 5/0 monofilament.

### Ultrasound and photo-acoustic imaging

Ultrasound and photo-acoustic imaging using a Vevo LAZR system (FUJIFILM VisualSonics Inc., Toronto, ON, Canada) and a real-time microvisualization LZ550 linear-array transducer (FUJIFILM VisualSonics Inc.) with a center frequency of 40 MHz was performed to detect hemoglobin oxygen saturation (sO_2_) within non-seeded and prevascularized Integra. Twenty-one days after implantation, the animals were anesthetized with 1.5% isoflurane and positioned in prone position on a heated stage. Sterile ultrasound gel was applied to avoid air interference with ultrasound coupling into the animal.

For three-dimensional high-resolution B-mode ultrasound and OxyHemo-mode photo-acoustic imaging, the scanhead was driven over the entire implant by a linear motor to acquire two-dimensional images at parallel and uniformly spaced, 150 μm-sized intervals. Oxy-Hemo-mode photo-acoustic images were recorded at 750 nm and 850 nm with a two-dimensional gain of 40 dB to detect sO_2_ within the samples^41,42^. Values were computed using the Vevo LAB 1.7.2. software (FUJIFILM VisualSonics Inc.).

### Trans- and epi-illumination stereomicroscopy

The implants and the surrounding skin were excised at day 21, placed under the stereomicroscope and digital images in TIF format were recorded. Using the software package ImageJ^43^ the background was subtracted and the images were converted into binary black and white images allowing the quantification of the vascular network. The vascularization (given in %) was defined as (vascularized area/total implant area) ∗ 100.

Planimetric analyses based on repetitive *in vivo* epi-illumination microscopy were performed to quantify the survival of the skin grafts. For this purpose, the anesthetized mice were placed under the stereomicroscope and digital images in TIF format were recorded. Using ImageJ, the survival of the skin grafts (given in %) was evaluated as follows: (Vital skin graft area/total skin graft area) ∗ 100.

### Experimental protocol

Ad-MVF of 8 GFP^+^ donor mice were isolated and seeded onto 8 Integra matrices. The matrices were implanted for 21 days into full-thickness skin defects on the skull of CD1 nu/nu mice. Eight non-seeded implants served as controls. *In vivo* imaging of the matrices was performed using epi-illumination stereomicroscopy (day 0, 3, 7, 14 and 21) and ultrasound/photo-acoustic imaging (day 21). At day 21, the animals were sacrificed and trans-illumination stereomicroscopy was performed. Finally, the specimen were processed for histological and immunohistochemical analyses.

In additional experiments, ad-MVF of 6 GFP^+^ donor mice were seeded onto Integra and the dermal substitutes were covered with STSG 10 days after implantation into skin defects of CD1 nu/nu mice. Six non-seeded, STSG-covered implants served as controls. The grafts were assessed by epi-illumination stereomicroscopy directly after implantation and at day 5. The animals were killed and the specimen were processed for histological and immunohistochemical analyses.

### Histology and immunohistochemistry

Formalin-fixed tissue samples were embedded in paraffin and cut into 3-mm thick sections. Sections were stained with hematoxylin and eosin (HE) or Sirius red for the visualization of mature collagen fibers (type I)^44^. With a BX60 microscope (Olympus, Hamburg, Germany) and the imaging software cellSens Dimension 1.11 (Olympus), the collagen content in relation to normal skin was assessed in 3 regions of interest (ROIs) of each sample.

Additional sections were stained with a monoclonal rat anti-mouse antibody against CD31 (1:100; dianova GmbH, Hamburg, Germany) and a polyclonal rabbit antibody against lymphatic vessel endothelial hyaluronan receptor-1 (LYVE-1; 1:200; Abcam, Cambridge, UK). A goat anti-rat IgG-Alexa555 antibody (1:100; Molecular Probes, Eugene, OR, USA) and a goat anti-rabbit IgG-Alexa555 antibody (1:200; Molecular Probes) served as secondary antibodies. Cell nuclei were stained with Hoechst 33342 (2 µg/mL; Sigma-Aldrich, Taufkirchen, Germany). The density of CD31^+^ blood and LYVE-1^+^ lymphatic vessels (given in mm^−2^) and the fraction of CD31^+^/GFP^+^ blood and LYVE-1^+^/GFP^+^ lymphatic vessels (given in %) were assessed within 5 ROIs of each implant.

To differentiate between GFP^+^ and GFP^-^ blood and lymphatic vessels, sections were stained with the mentioned primary and secondary antibodies against CD31 and LYVE-1 and with a polyclonal goat anti-GFP antibody (1:100; Rockland, Limerick, PA) to enhance GFP fluorescence. A biotin-labeled donkey anti-goat IgG antibody (1:15; Jackson ImmunoResearch, Baltimore, MD) was used as secondary antibody and was detected by fluorescein labeled-streptavidin (1:50; Vector Labs, Burlingame, CA). For this purpose, sections were placed in Coplin jars with 0.05% citraconic anhydride solution (pH 7.4) for 1 h at 98 °C and incubated overnight at 4 °C with the primary antibody, followed by the secondary antibody at 37 °C for 2 h. Finally, the fraction of GFP^+^ and GFP^-^ blood and lymphatic vessels was assessed.

For the immunohistochemical assessment of STSG epithelialization, sections were stained with a polyclonal rabbit antibody against cytokeratin (1:100; Abcam). As secondary antibody a biotinylated goat anti-rabbit IgG antibody (ready-to-use; Abcam) was used, which was detected by peroxidase-labeled-streptavidin (1:50; Sigma-Aldrich). 3-Amino-9-ethylcarbazole (Abcam) was used as chromogen. The sections were counterstained with Mayer’s hemalum solution (Merck, Darmstadt, Germany).

### Statistics

Data were tested for normal distribution and equal variance. Differences between groups were analyzed using the unpaired Student *t* test (SigmaPlot; Systat Software Inc, San Jose, CA, USA). All values are expressed as mean ± standard error of the mean (SEM). Statistical significance was accepted for values of *P* < 0.05.

### Data availability

The datasets generated and/or analyzed during the current study are available from the corresponding author on reasonable request.
